# Editorial: Optical Approaches to Capture Plant Dynamics in Time, Space, and Across Scales

**DOI:** 10.3389/fpls.2018.00791

**Published:** 2018-06-11

**Authors:** Eetu Puttonen, Alexander Bucksch, András Zlinszky, Norbert Pfeifer

**Affiliations:** ^1^Remote Sensing and Photogrammetry, Finnish Geospatial Research Institute, Kirkkonummi, Finland; ^2^Center of Excellence in Laser Scanning Research, Masala, Finland; ^3^Warnell School of Forestry and Natural Resources, University of Georgia, Athens, GA, United States; ^4^Institute of Bioinformatics, University of Georgia, Athens, GA, United States; ^5^Department of Plant Biology, University of Georgia, Athens, GA, United States; ^6^Balaton Limnological Institute, Centre for Ecological Research, Hungarian Academy of Sciences, Tihany, Hungary; ^7^Department of Geodesy and Geoinformation, Technische Universität Wien, Vienna, Austria

**Keywords:** plant growth, plant development, geometric changes in plants, seasonal changes in vegetation structure, change detection

The quest to decipher the phenotype to genotype relationships involves the quantification of plant adaption to the environment at various scales to solve some of the world's most pressing problems (Bucksch et al., 2017). The role of phenotype to genotype relationships within initiatives to increase crop yields for food, fiber, and fuel and to improve prediction of future environmental conditions (IPCC, 2014) is central to the function and well-being of societies world-wide. Quantifying plant morphology over time captures the dynamic structure and function relationships of how plants interact with and respond to environmental stimuli (Balduzzi et al., 2017). A deeper understanding of plant adaption can be achieved if technologies to monitor plants across spatial and biological scales are further developed. Spanning biological scales from the community over the organismal down to the molecular scale is inherently coupled to spatial scales. A wide variety of technological concepts are facilitating the revival of the science of plant morphology and anatomy (Ledford, 2018).

Over the last decades, optical imaging and remote sensing developed the fundamental working tools to monitor and quantify our environment and plants in particular. Satellite imaging increased its spatial, temporal, and spectral resolution to levels where individual trees in forests can be partly identified. However, it remains a challenge to develop pipelines that quantify the traits of plants and their processes from the level of plant populations down to the detail obtained with microscopy. The reason for the pipeline challenge is the heterogeneity of the obtained data ranging from unorganized point clouds over surface models to rasterized imaging and hyperspectral data. For example, airborne methods increased their importance in ecology by measuring aggregate tree traits such as crown width or stem diameter to study community composition (Wieser et al., 2017). However, combining airborne and high-resolution terrestrial data is non-trivial despite improved automatization and higher resolutions of measured plant traits. Unmanned aerial vehicles (UAV) equipped with cameras and multispectral sensors allow the fusion of temporal, spatial, and spectral data to record plant dynamics on ecosystem level at resolutions that also allow the quantification of individual plant morphology.

In particular, the advances in algorithms to handle heterogenous data of various sensors revealed details of plant adaptation to discover the genes controlling adaptation mechanisms. The wealth of options presents a new challenge in testing and selecting an appropriate approach that scales well with the research questions. Making modern technology applicable to the hypothesis driven science process requires more than learning a few techniques. In the light of a particular hypothesis it requires also resources to acquire, build, and refactor equipment and software. Here, a wider view to different research disciplines and how they utilize their state-of-the-art techniques can be most beneficial in demonstrating potential use cases. Ideally, this will lead to joint use of best practices in technology innovation and discovery in the plant sciences.

The Topic received a diverse set of 11 original research manuscripts that cover a wide array of applications across different scales. On the spatial scale, Scharr et al. present a fast imaging approach to perform volumetric 3D carving of plant roots on submillimeter level. Sun et al. apply LiDAR for high-throughput phenotyping on cotton plants while Lin et al. use LiDAR mapping to investigate how Hallé's models of tree architecture reflect in real data. Xu et al. apply Structure-from-Motion to images collected with unmanned aerial vehicle in cotton fields to detect the exact flowering time and the precise locations of cotton balls using convolutional neural networks. Swetnam et al. link individual plant morphology to plant populations by fusing point clouds collected with ground-based, UAV mounted, and airborne systems to study different structural states of a dryland ecosystem.

Temporal dynamics were quantified by Zlinszky et al. who measured variation in nocturnal branch movements between different species with short interval LiDAR. Similarly, Herrero-Huerta et al. studied the movement of leaves in *Calathea roseopicta* under different lighting conditions with a LiDAR system. The outcome of these investigations challenges our view of plants as passive, static organisms, and shows that they are capable of short-term changes in shape at the whole plant level.

While not completely non-invasive, Doan et al. published a first low-cost imaging system to detect formation of free amino acid groups on maize roots to elucidate exudate responses. In doing so, a special, ninhydrin-injected paper was monitored over a 3-week growth period. Jiang et al. reported an application using low-cost consumer RGB-D cameras to monitor and quantify the growth of cotton canopies over a 3-month period.

In addition, two manuscripts harnessed spectral information to study dynamic responses in plant leaves outside the visible region. Cen et al. detected photosynthetic fingerprints of citrus Huanglongbing (so-called “greening”) disease with fluorescence spectroscopy by combining imaging of specific fluorescence properties with advanced image analysis. Junttila et al. estimated leaf equivalent water thickness using combinations of different LiDAR wavelengths. Their results showed significant correlation with the standard laboratory reference measurements. These findings demonstrate the significant potential in expanding multi-wavelength laser scanning measurements to whole forests to accurately monitor ecophysiological parameters.

Our Research Topic proves that optical technologies have reached a level of precision, reliability and detail that is sufficient to study minute physiological processes in plants. In addition to documenting plant status at various levels, imaging, and scanning methods allow following processes of adaptation, movement or disease in real time as they happen. In addition to serving as early detection systems (Cen et al.; Junttila et al.) for management purposes, these methods allow new insights into plant function. One particular example for this is the observed short-term periodicity in plant movement and trunk diameter, which suggests a new approach to water transport in trees (Zlinszky and Barford, 2018).

The Research Topic covered a plethora of methodological approaches as suggestions for best practices in the light of a particular research question. As such, the Research Topic collected papers that demonstrate how technology development and scientific discovery in the plant sciences can accelerate each other toward deciphering the adaption of plants as part of the quest to reveal the phenotype to genotype map. The future challenge will be to extend the multi-scale measurement of static traits to multi-scale measurement of chemical processes within and between plants. For example, how do diseases or ripening processes affect the community structure or how does microclimate fluctuation of single plants affect overall edible yield? How do shape changes in canopies relate to the hydraulic properties at tissue level? We believe that the optical methods in this Research Topic will become openly available such that all researchers across the plant sciences will be capable to perform research at this exciting frontier.

## Author contributions

All authors listed have made a substantial, direct and intellectual contribution to the work, and approved it for publication.

### Conflict of interest statement

The authors declare that the research was conducted in the absence of any commercial or financial relationships that could be construed as a potential conflict of interest.
